# Hospital Meetings

**Published:** 1903-05-23

**Authors:** 


### HOSPITAL MEETINGS.

### NATIONAL SANATORIUM FOR CONSUMPTION, BOURNEMOUTH.

The annual general meeting of Governors was held in London on the 14th inst., the Right Hon. Arthur F. Jeffreys, M.P., in the chair.

The annual report of the committee for the year 1902 was placed before the Governors with the statement of accounts showing a deficit of £109, which, added to the existing debt of £431, amounts to a total deficiency of £540. A special appeal is being made in order to put the Institution in a better financial position.

It is pointed out that the benefits of the National Sanatorium are not limited to the patients who enter it, but extend to the public generally, for every case in which the disease of consumption is arrested, removes a source of infection to others. Moreover, each patient is taught the laws of hygiene and the means of preventing the spreading of the disease to others and the re-infection of themselves.

The report further states that during the year his Majesty the King was graciously pleased to send a donation of £100.

The retiring members of committee were re-elected. A hearty vote of thanks was passed to the physicians, the surgeons, and the dental surgeon for their services during the past year.

